# Invited commentary: Lymph node yield independently predicts local recurrence in papillary thyroid cancer

**DOI:** 10.1002/wjs.12396

**Published:** 2024-10-31

**Authors:** Fausto F. Palazzo

**Affiliations:** ^1^ Hammersmith Hospital ‐ Endocrine Surgery London UK

Factors that adversely influence the risk of recurrence after the initial treatment of papillary thyroid cancer (PTC) include the disease stage, the presence of aggressive histological subtypes, and adverse biomarkers.^1^ Inevitably, the risk of PTC recurrence is also affected by the adequacy of the medical care provided. Normally the assessment of the quality of cancer care consists in comparing the outcomes achieved between services. However, the often forgiving biology and a 5‐year survival of PTC of over 98% coupled with the complexity of the variables that influence thyroid cancer outcomes mean that the focus in PTC quality assurance has been on the structure and process of care rather than cancer survival. An example of a measure of the structure of care is the repeatedly proven correlation between higher volume thyroid surgeons and their institutions and lower morbidity.^2^ It has not been proven that the PTC survivals are worse in low volume thyroid surgery practices for the reasons above, but this important structure measure has, with variable success, driven the centralization of thyroid surgery.

Yang and colleagues' study on what defines adequate lymph node surgery is an analysis of the process in cancer care and the consequences if the treatment is not optimal.^3^ The rate of PTC local persistence or recurrence is a measure of the quality of the surgical process that in some cancers might measurably affect outcome but in PTC probably due to the often forgiving biological nature of the disease acts only as a surrogate predictor of clinical long‐term cancer outcome.^4^ Lymph node yield impacts on the tumor stage in cancers staged using the TNM system and therefore guides the indication for adjuvant treatment—a typical example of this would be colorectal cancer. However, in squamous cell carcinoma of the head and neck, nodal yield has also been demonstrated to impact also on patient survival.^5^ In thyroid cancer, whilst local recurrence is more common if the nodal yield is low, to demonstrate a worse survival profile effect would require follow‐up periods that exceed entire medical careers.

The identification of surgical quality assurance parameters in thyroid cancer care presents many challenges, and nodal yield in PTC reflects this well:

Firstly, given that a randomized controlled trial would be impractical and/or unethical, retrospective case series tend to be used instead. PTC is a relatively uncommon disease, and whilst nodal micrometastases are ubiquitous, clinically relevant nodal disease is less common. Therefore, decades of data collection are required and with this the potential inconsistencies of reporting that may occur over time. For example, the way in which recurrence is detected has changed over 3 decades with ultrasensitive thyroglobulin assays and higher resolution ultrasound that will increase recurrence detection. We may today be unveiling what was previously subclinical recurrence that would have gone unnoticed and untreated possibly without any proven detriment to the patient. The lymph node yield may be affected by the surgical intent of the procedure: prophylactic central nodal dissection may have a lower yield than one performed with therapeutic intent. Furthermore lymph node yield can simply be a reflection of the enthusiasm of the reporting pathologist.

The second challenge is whether the findings, which in this paper appear to be as robust as one can hope in a study of its type, can be interpreted in the same way in all environments. Practices vary regarding whether the central nodal dissection always includes level VII, and whether the lateral nodal dissection always includes level VB and these can significantly influence the lymph node yield.

However, the third and most important question is what do we do with this information that suggests that a lymph node count below a threshold increases the risk of recurrence? Do we adopt minimum standards or aspirational higher targets? Who after all wants to be treated by the half of us that are *below average* surgeons? And how can these higher standards be implemented?

If the minimum numbers of thyroid, parathyroid, and adrenal procedures agenda has taught us anything it is that it can be very difficult to enforce structural and process change in surgical care. We do know however that the act of measuring healthcare performance seems to improve outcomes, possibly due to the Hawthorne effect.^6^ The submission of surgical practice data to national or international registries provides an opportunity for surgeons to reflect on personal outcomes and may nurture an ethos of self‐improvement, especially if there is a supportive environment and educational programs are available. Studies like that of Yang and colleagues provide a spotlight on the importance of surrogate outcome benchmarks, underline the value of training and the imperative of collective self‐regulation which in the long term is most likely to improve thyroid cancer care.

## AUTHOR CONTRIBUTIONS

**Fausto F. Palazzo**: Conceptualization; writing – original draft; writing – review & editing.
